# Comparison of Delta-Shape Anastomosis and Extracorporeal Billroth I Anastomosis after Laparoscopic Distal Gastrectomy for Gastric Cancer: A Systematic Review with Meta-Analysis of Short-Term Outcomes

**DOI:** 10.1371/journal.pone.0162720

**Published:** 2016-09-15

**Authors:** Geng-yuan Hu, Feng Tao, Ke-wei Ji, Wei Wang

**Affiliations:** Department of Gastrointestinal Surgery, Shaoxing People’s Hospital, Shaoxing Hospital of Zhejiang University, Shaoxing, China; Fu Jen Catholic University, TAIWAN

## Abstract

**Objective:**

The aim of this systematic review and meta-analysis is to evaluate the safety and relative benefits of delta-shape anastomosis (DA) by comparing to conventional laparoscopy-assisted distal gastrectomy with Billroth I gastroduodenostomy (LADG BI).

**Methods:**

Studies and relevant literature regarding DA versus LADG BI were searched in the electronic databases. Operation time, postoperative complications, estimated blood loss, number of retrieved lymph nodes, time to first flatus, time to oral intake, length of postoperative hospitalization in DA and LADG BI were pooled and compared using meta-analysis. Weighted mean differences (WMDs) and odds ratios (ORs) were calculated with 95% confidence intervals (CIs) to evaluate the effect of DA.

**Results:**

Eight studies of 1739 patients were included in the meta-analysis. Compared with LADG BI, DA had shorter postoperative hospitalization (WMD = -0.47, 95%CI: -0.69 to -0.25, P<0.01), less blood loss (WMD = - 25.90, 95%CI: -43.11 to -8.70, P<0.01), shorter time to oral intake (WMD = -0.25, 95%CI: -0.49 to -0.01, P = 0.04), and more retrieved lymph nodes (WMD = 1.36, 95%CI: 0.30 to 2.43, P = 0.01). Operation time (WMD = -0.07, 95%CI -15.58 to 15.43, P = 0.99), overall postoperative complication rate (OR = 1.05, 95%CI: 0.74 to 1.49, P = 0.63), surgical complication rate (OR = 1.02, 95%CI: 0.70 to 1.49, P = 0.90), nonsurgical complication rate (OR = 1.21, 95%CI: 0.54 to 2.72, P = 0.64), leakage rate (OR = 2.54, 95%CI: 0.92 to 7.01, P = 0.07), stricture rate (OR = 0.36, 95%CI: 0.09 to 1.44, P = 0.15), wound complication rate (OR = 0.71, 95%CI: 0.33 to 1.55, P = 0.39), time to first flatus (WMD = -0.10, 95%CI: -0.27 to 0.07, P = 0.26), and proximal surgical margin (WMD = -0.25, 95%CI: -1.14 to 0.65, P = 0.59) was not statistically different.

**Conclusion:**

Compared with LADG BI, DA is a safe and feasible procedure, with significantly reduced blood loss, time to oral intake, and postoperative hospitalization.

## Introduction

Radical gastrectomy remains the main managements of gastric cancer. Three methods of gastrointestinal tract reconstruction, including the Billroth I (BI) gastroduodenostomy, Billroth II (BII) gastrojejunostomy and Roux-en-Y (R-Y) gastrojejunostomy, are commonly used after distal gastrectomy. Among these methods, the BI anastomosis is especially preferred due to technical simpleness and physiological advantages of allowing food to pass through the duodenum. Besides, postoperative endoscopic examination for biliary tract disorders after BI anastomosis is thought to be easier when compared with that after Billroth II or R-Y anastomosis.

Laparoscopic gastrectomy (LG) has gradually matured and been accepted as a notable alternative to open surgery in the management of gastric cancer [1–3]. More than 90% of patients with early gastric cancer can survive following laparoscopic radical gastrectomy [4–6]. For this population, more comfortable perioperative experience and better postoperative quality of life (QoL) are important goals as well. Usually, surgeons completed the lymph node dissection with laparoscopic techniques and performed gastrointestinal tract reconstruction through the mini-laparotomy, namely laparoscopy-assisted gastrectomy. Advancements in less invasive techniques are ongoing, and many surgeons are attempting to perform totally laparoscopic gastrectomy, which is expected to achieve less invasiveness and better postoperative QoL [7–9]. However, challenges lie in the intracorporeal hand-sewn technique. In 2002, Kanaya et al. described a novel technique named the delta-shaped anastomosis (DA) [10], which was derived from the application of the functional end-to-end technique[11]. The DA completes BI anastomosis just with laparoscopic linear staplers and greatly facilitates intracorporeal BI anastomosis, which gradually gained popularity in Japan, Korea and China [12–14]. Given the critical roles of anastomosis procedure on surgical outcomes, which were still unsettled in DA, we conducted this meta-analysis to clarify the safety and relative benefits of DA by comparing to LADG with Billroth I gastroduodenostomy (LADG BI).

## Methods

### Literature search

A systematic search was made using PubMed, ISI web of knowledge, Scopus and Embase, from January 2002 to March 2016, to retrieve all published articles comparing DA and LADG BI. The search term was (“delta-shaped anastomosis” or “intracorporeal Billroth I anastomosis”) and (“laparoscopic gastrectomy” or “minimally invasive gastrectomy”) and (“gastric cancer” or “gastric adenocarcinoma” or “gastric neoplasms”). The “related articles”, “similar articles” was also reviewed to broaden the search. The language of publications was restricted in English and Chinese.

### Eligibility criteria

All the publications retrieved were included if they were 1) comparing DA with LADG BI; 2) reporting at least 20 patients in each study group; 3) investigating all the primary outcomes in our standardized questionnaires; and 4) in the event that duplication of data was observed, more recent studies or those with larger sample sizes were preferentially considered. A manual search of the references of all retrieved articles was also carried out to identify publications for possible inclusion.

### Exclusion criteria

The following studies or data were excluded: 1) Abstracts, letters, editorials, expert opinions, reviews without original data and case reports; 2) the outcomes and parameters of patients were not clearly reported; 3) it was impossible to extract the appropriate data of the primary outcomes from the published results; and 4) there was an overlap between authors or centers in the published literature.

### Data extraction and synthesis

Two reviewers independently undertook literature searches, screened abstracts, and assessed articles met eligibility criteria. The quality of the included studies was assessed using the Newcastle-Ottawa Scale (NOS) (http://www.ohri.ca/programs/clinical_epidemiology/oxford.asp). Studies achieving six or more scores were considered to be of high quality and were included in the meta-analysis. Reviewers extracted the following parameters from each study: (1) author and study period; (2) study population characteristics, study design; (3) number of patients operated on with each technique; and (4) preoperative data, intraoperative data and postoperative data. Discrepancies between the two reviewers were resolved by discussion with all co-authors and consensus was reached.

### Outcomes of interest

The following outcomes were used to compare the two operative techniques: (1) primary outcomes, which referred to operation time, postoperative stay, overall postoperative complication rate, surgical complication rate, nonsurgical complication rate, anastomotic leakage rate, anastomotic stricture rate, wound complications rate; (2) secondary outcomes, which included estimated blood loss (EBL), time to first flatus, time to oral intake earlier, number of retrieved lymph nodes and proximal surgical margins. The surgical and nonsurgical complication was defined as Jung et al. [15].

### Statistical analysis

The meta-analysis was performed using the Review Manager software, version 5.3, provided by the Cochrane Collaboration (http://tech.cochrane.org/revman/download). We analyzed dichotomous variables using the estimation of odds ratios (ORs) with 95% confidence interval (95% CI) and continuous variables using weighted mean differences (WMDs) with a 95% CI. According to the Higgins I^2^ statistic, heterogeneities <25%, 25% to 50%, and >50% were defined as low, moderate, and high, respectively [16]. A fixed-effects model was used for studies with low or moderate statistical heterogeneity [17]. Otherwise, a random-effects model was used for studies with high statistical heterogeneity [18]. A subgroup analysis of studies more than 50 cases in both DA and LADG BI was conducted. An estimate of potential publication bias was carried out using the funnel plot. P <0.05 was considered statistically significant.

## Result

### Selected studies characteristics

**Fig 1:**
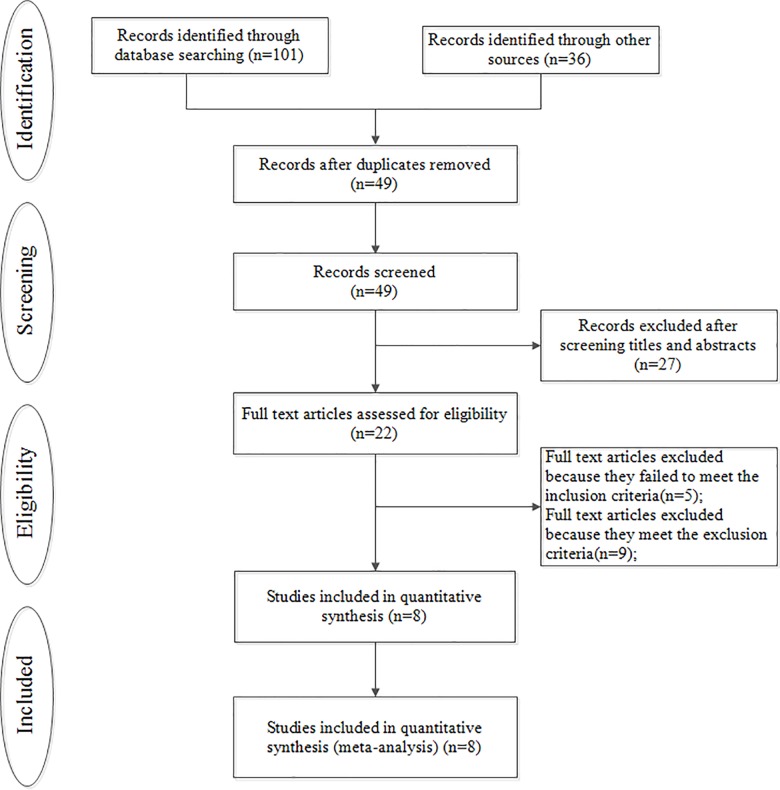
Flow chart of the studies included in the meta-analysis.

After selection, eight studies were eventually included in our research [13,19–25] (Fig 1). In total, 755 patients treated with DA and 984 patients treated with LADG BI were included in the analyses (Table 1). All the patients were from Eastern Asia, including Korea, Japan and China. The majority of patients underwent DA were with early gastric cancer (520 out of 755). All of the eight studies were retrospective, nonrandomized. All eight studies were considered to be of adequate quality for the meta-analysis according to NOS assessment (score >5 points) (Table 2).

**Table 1:** Main characteristics of all studies included in the meta-analysis.

Study	Study period	Country	Study design	Group	Case number	Mean age	Gender (M/F)	BMI	EGC/AGC	Matching criteria
Kinoshita et al.	2007~2009	Japan	R	DA	42	64.7	25/17	23.1	38/4	abcdgh
				LADG BI	41	68.4	30/11	22.8	37/4	
Kim MG et al.	2009~2010	Korea	R	DA	239	56.6	155/84	24	204/35	abfe
				LADG BI	328	55.4	198/130	23.1	312/16	
Kim DG et al.	2009~2012	Korea	R	DA	60	58.3	37/23	23.4	49/11	abceg
				LADG BI	106	55.8	69/37	23.1	90/16	
Wang et al.	2013~2014	China	R	DA	50	64	34/16	23	9/41	abceh
				LADG BI	43	61.2	28/15	22.3	5/38	
Lee et al.	2004~2011	Korea	R	DA	138	62.4	87/51	24.2	94/6	dfgh
				LADG BI	100	56	47/53	22.6	127/11	
Jeong et al.	2013~2014	Korea	R	DA	42	58.4	22/20	24.8	42/0	bcdfgh
				LADG BI	179	62.7	114/65	24.1	167/12	
Lin et al.	2011~2014	China	R	DA	143	60.1	100/43	22.3	48/95	abcg
				LADG BI	143	59.4	102/41	23.5	53/90	
Park et al.	2013~2014	Korea	R	DA	41	61.7	23/18	24.3	36/5	abcdfgh
				LADG BI	44	62.2	24/20	23.4	42/2	

M, male; F, female; EGC, early gastric cancer; AGC, advanced gastric cancer; R retrospective; a, age; b, gender; c, BMI; d, comorbidity; e, ASA; f, tumor size; g, tumor stage; h, extend of lymph node dissection.

**Table 2:** Newcastle-Ottawa Scale assessment of pooled studies.

Study	Selection				Comparability	Outcomes			Total
	Representativeness of exposed cohort	Selection of nonexposed cohort	Ascertainment of exposure	Outcome not present at the start of the study		Assessment of outcomes	Length of follow-up	Adequacy of follow-up	
Kinoshita et al.	*	*	*	*	**	*	*	*	*********
Kim MG et al.	*	*	*	*	*	*			******
Kim DG et al.	*	*	*	*	**	*	*	*	*********
Wang et al.	*	*	*	*	**	*			******
Lee et al.	*	*	*	*	*	*	*	*	*********
Jeong et al.	*	*	*	*	**	*			******
Lin et al.	*	*	*	*	*	*			******
Park et al.	*	*	*	*	**	*	*	*	*********

*, one score.

### Primary outcomes

**Fig 2:**
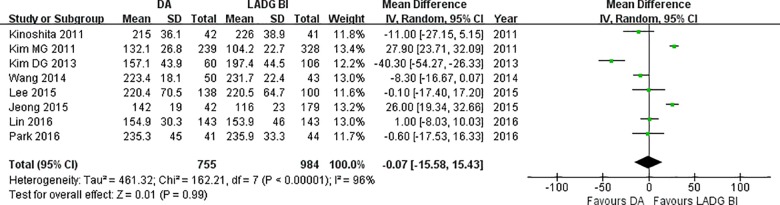
Forest plots of operation time, DA vs. LADG BI.

**Fig 3:**
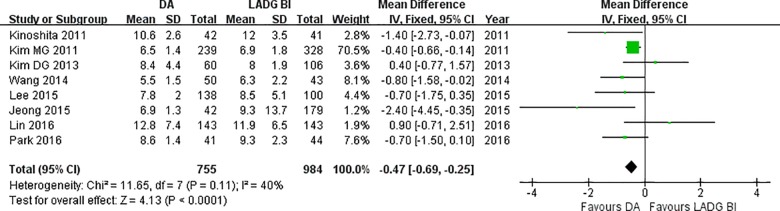
Forest plots of length of hospitalization, DA vs. LADG BI.

**Fig 4:**
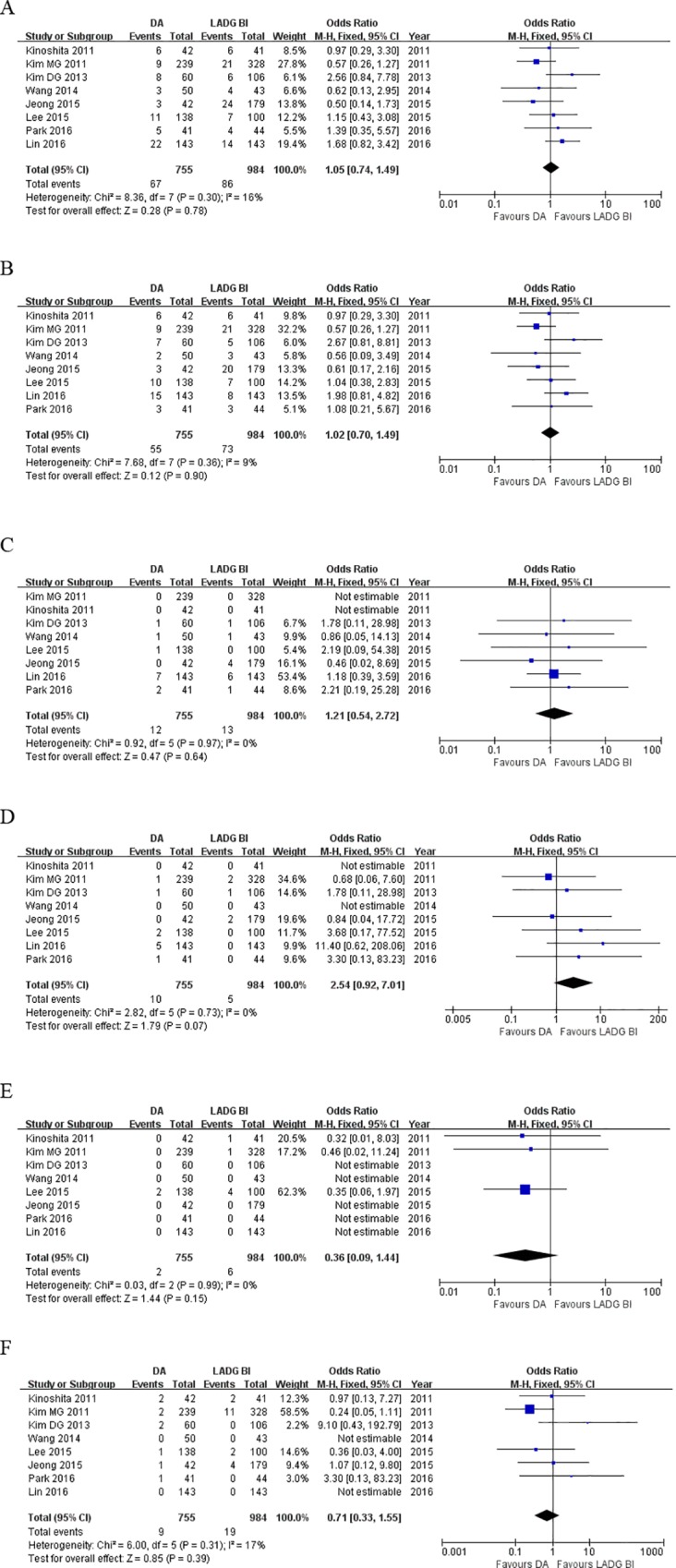
Forest plots of postoperative complication, DA vs. LADG BI. (A) overall postoperative complication, (B) surgical complication, (C) nonsurgical complication, (D) anastomotic leakage, (E) anastomotic stricture, and (F) wound complication.

As showed in Fig 2, there was no significant difference between DA and LADG BI regarding the operation time (WMD = -0.07, 95%CI -15.58 to 15.43, P = 0.99). Patients underwent DA had a shorter postoperative stay than those underwent LADG BI (WMD = -0.47, 95%CI: -0.69 to -0.25, P<0.01) (Fig 3). Sixty-seven out of 755 patients in the DA group and 86 out of 984 patients in LADG BI group had postoperative morbidities (Table 3). There was no significant difference between two groups (OR = 1.05, 95%CI: 0.74 to 1.49, P = 0.63) (Fig 4A). The pooled effect showed no significant difference between DA and LADG BI in surgical complication rate (OR = 1.02, 95%CI: 0.70 to 1.49, P = 0.90) (Fig 4B) and nonsurgical complication rate (OR = 1.21, 95%CI: 0.54 to 2.72, P = 0.64) (Fig 4C). Regarding the anastomotic leakage, no statistically significant difference was observed (DA *vs*. LADG 10/755 *vs*.5/984; OR = 2.54, 95%CI: 0.92 to 7.01, P = 0.07) (Fig 4D). Two out of 755 patients in the DA group and 6 out of 984 patients in the LADG BI group suffered anastomotic stricture with no significant difference between two groups (OR = 0.36, 95%CI: 0.09 to 1.44, P = 0.15) (Fig 4E). DA had similar wound complications rate compared with LADG BI (OR = 0.71, 95%CI: 0.33 to 1.55, P = 0.39) (Fig 4F).

**Table 3:** Details of postoperative morbidities in pooled studies.

Study	Group	Case number	Complication
Kinoshita et al	DA	42	Intra-abdominal abscess×2, Wound complications×2, Others×2
	LADG BI	41	Anastomotic stenosis×1, Bleeding×1, Intra-abdominal abscess×1, Wound complications×2, Others×1
Kim MG et al	DA	239	Anastomotic leakage×1, Bleeding×2, Intra-abdominal abscess×3 Wound complications×2, Others×1
	LADG BI	328	Anastomotic leakage×2,Anastomotic stenosis×1, Bleeding×3, Intra-abdominal abscess×4, Wound complications×11
Kim DG et al	DA	60	Anastomotic leakage×1, Delayed gastric emptying×2, Intra-abdominal abscess×2, Wound complications×2, Others×1
	LADG BI	106	Anastomotic leakage×1, Intra-abdominal abscess×4, Others×1
Wang et al	DA	50	Delayed gastric emptying×1, Others×2
	LADG BI	43	Bleeding×1, Others×3
Lee et al	DA	138	Anastomotic leakage×2, Anastomotic stenosis×2, Bleeding×1, Wound complications×1, Others×4
	LADG BI	100	Anastomotic stenosis×4, Wound complications×2, Others×1
Jeong et al	DA	42	Bleeding×1, Delayed gastric emptying×1, Wound complications×1
	LADG BI	179	Anastomotic leakage×2,Anastomotic stenosis×1, Bleeding×3, Intra-abdominal abscess×4, Wound complications×11
Lin et al	DA	143	Anastomotic leakage×5, Bleeding×1, Delayed gastric emptying×1, Intra-abdominal abscess×3, Others×12
	LADG BI	143	Bleeding×2, Delayed gastric emptying×1, Intra-abdominal abscess×3, Others×8
Park et al	DA	41	Anastomotic leakage×1, Wound complications×1, Others×3
	LADG BI	44	Anastomotic leakage×1, Intra-abdominal abscess×1, Others×2

### Secondary outcomes

**Fig 5:**
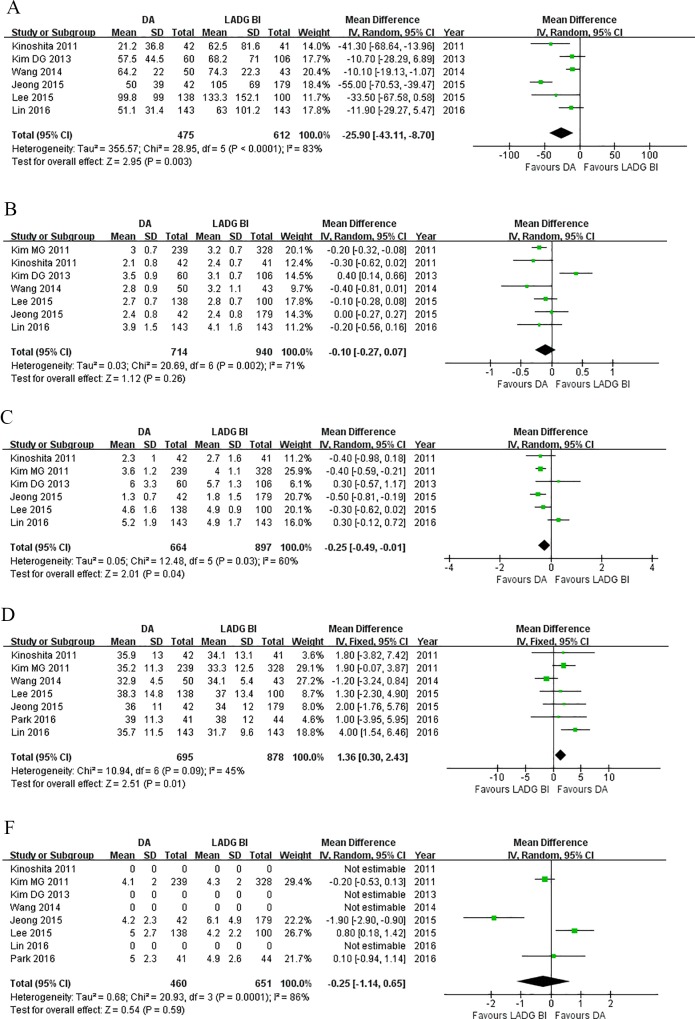
Forest plots of secondary outcomes, DA vs. LADG BI. (A) EBL, (B) time to first flatus, (C) time to first oral taking, (D) number of retrived lymph nodes, and (E) proximal surgical margin.

Secondary outcomes were summarized in Table 4 and Fig 5. Six out of the included studies reported EBL in both groups [13,19,21–24]. A significant reduction in blood loss was observed in the DA compared to LADG BI (WMD = - 25.90, 95%CI: -43.11 to -8.70, P<0.01).

**Table 4:** Meta-analyses results for DA vs. LADG BI.

Outcomes	Pooled studies	Sample size	Pooled effect	Pooled estimates	95% CI	P value	I^2^
EBL	6	1087	Random	WMD -25.90	-43.11, -8.70	<0.01	83%
Time to first flatus	7	1654	Random	WMD -0.10	-0.27, 0.07	0.26	71%
Time to first oral taking	6	1561	Fixed	WMD -0.25	-0.49, -0.01	0.04	60%
Retrived lymph nodes	7	1573	Fixed	WMD 1.36	0.30, 2.43	0.01	45%
Proximal surgical margin	4	1111	Random	WMD -0.25	-1.14, 0.65	0.59	86%

EBL, estimated blood loss; WMD, weighted mean difference; OR, odds ratio.

As for the postoperative recovery, DA groups had similar time to first flatus (WMD = -0.10, 95%CI: -0.27 to 0.07, P = 0.26). Patients in the DA group started oral intake earlier than those in LADG BI group (WMD = -0.25, 95%CI: -0.49 to -0.01, P = 0.04).

DA retrieved more lymph nodes as compared with LADG BI (WMD = 1.36, 95%CI: 0.30 to 2.43, P = 0.01). Proximal surgical margins were equivalent between two groups (WMD = -0.25, 95%CI: -1.14 to 0.65, P = 0.59).

### Nutritional status and postgastrectomy symptoms

Four pooled studies reported the postoperative nutritional status and postgastrectomy symptoms [13,19,22,25], as showed in Table 5. Lee et al. reported DA group had more food intake [22]. Park et al. reported DA group maintained higher albumin postoperatively [25]. Kim et al. and Park et al. found more patients in the DA group suffered reflux [13,25]. No difference was revealed between the DA group and LADG BI group regarding nausea, dyspepsia, diarrhea and dumping syndrome.

**Table 5:** details of postoperative nutritional status and postgastrectomy symptoms.

Study	Length of follow-up	Nutritional status				Total lymphocyte count	Assesment terms of gastrointestinal symptoms	Postgastrectomy symptoms				
		Food intake	Body weight	Serum albumin	Total cholesterol			Reflux	Nausea	Dyspepsia	Diarrhea	Dumping syndrome
Kinoshita et al.	3 months		NS	NS	NS		questionnaire	NS	NS	NS	NS	NS
Kim DG et al.	3 months		NS				questionnaire	↓		NS	NS	NS
Lee et al.	46 months (mean)	↑	NS			NS	questionnaire and endoscopy examination	↓		NS		NS
Park et al.	1 year		NS	↑	NS	NS	questionnaire	NS	NS		NS	

NS, no significance; ↓, worse; ↑, better.

### Subgroup analysis

Subgroup analysis of the pooled studies was performed to evaluate whether the pooled primary outcomes and secondary outcomes altered in different case volume subgroup. Subgroup analysis regarding the surgical outcomes had similar results as above (Table 6).

**Table 6:** Subgroup analyses results for DA vs. LADG BI.

Outcomes	Pooled studies	Sample size	Pooled estimates	95% CI	P value
Operation time	8	1739	WMD -0.07	-15.58, 15.43	0.99
≤50 cases	4	482	WMD 2.01	-19.32, 23.34	0.85
˃50 cases	4	1257	WMD -2.43	-30.81, 25.96	0.87
Length of hospitalization	8	1739	WMD -0.47	-0.69, -0.25	<0.01
≤50 cases	4	482	WMD -0.94	-0.60, -0.10	<0.01
˃50 cases	4	1257	WMD -0.35	-1.44, -0.44	<0.01
Overall postoperative complication	8	1739	OR 1.05	0.74, 1.49	0.78
≤50 cases	4	482	OR 1.05	0.41, 1.50	0.46
˃50 cases	4	1257	OR 1.19	0.78, 1.81	0.41
Surgical complication	8	1739	OR 1.21	0.54, 2.72	0.64
≤50 cases	4	482	OR 1.01	0.24, 4.25	0.99
˃50 cases	4	1257	OR 1.32	0.49, 3.52	0.58
Nonsurgical complication	8	1739	OR 1.02	0.70, 1.49	0.90
≤50 cases	4	482	OR 0.78	0.38, 1.57	0.48
˃50 cases	4	1257	OR 1.15	0.73, 1.81	0.54
Anastomotic leakage	8	1739	OR 2.54	0.92, 7.01	0.07
≤50 cases	4	482	OR 1.64	0.22, 12.16	0.63
˃50 cases	4	1257	OR 2.90	0.87, 9.66	0.08
Anastomotic stricture	8	1739	OR 0.36	0.09, 1.44	0.15
≤50 cases	4	482	OR 0.32	0.01, 8.03	0.49
˃50 cases	4	1257	OR 0.38	0.08, 1.71	0.21
Wound complication	8	1739	OR 0.71	0.33, 1.55	0.39
≤50 cases	4	482	OR 1.29	0.35, 4.77	0.70
˃50 cases	4	1257	OR 0.53	0.20, 1.41	0.20
EBL	6	1087	WMD -25.90	-43.11, -8.70	<0.01
≤50 cases	3	397	WMD -34.72	-67.60, -1.85	0.04
˃50 cases	3	690	WMD -13.89	-25.51, -2.27	0.02
Time to first flatus	7	1654	WMD -0.10	-0.27, 0.07	0.26
≤50 cases	3	397	WMD -0.20	-0.45, 0.04	0.11
˃50 cases	4	1257	WMD -0.03	-0.28, 0.21	0.8
Time to first oral taking	6	1561	WMD -0.25	-0.49, -0.01	0.04
≤50 cases	2	304	WMD -0.48	-0.75, -0.21	<0.01
˃50 cases	4	1257	WMD -0.12	-0.47, 0.23	0.51
Retrived lymph nodes	7	1573	WMD 1.36	0.30, 2.43	0.01
≤50 cases	4	482	WMD -0.13	-1.74, 1.49	0.88
˃50 cases	3	1091	WMD 2.50	1.09, 3.92	<0.01
Proximal surgical margin	4	1111	WMD -0.25	-1.14, 0.65	0.59
≤50 cases	2	306	WMD -0.91	-2.87, 1.05	0.37
˃50 cases	2	805	WMD 0.26	-0.71, 1.24	0.60

EBL, estimated blood loss; WMD, weighted mean difference; OR, odds ratio.

### Publication bias

**Fig 6:**
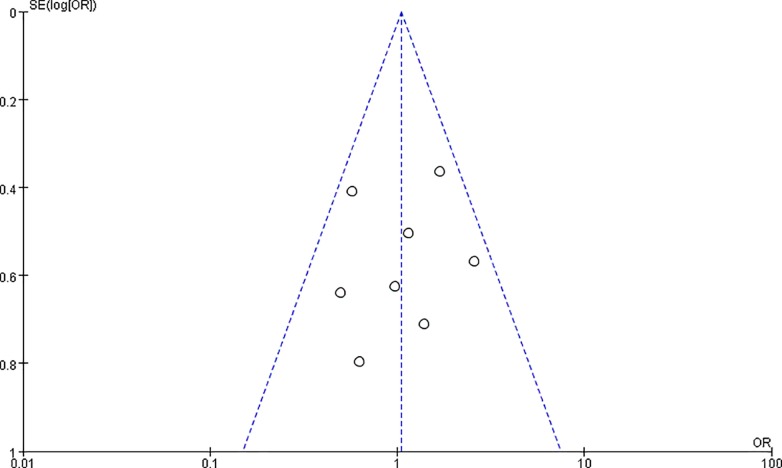
Funnel plots of postoperative complication.

A funnel plot was constructed for the overall postoperative complications and showed symmetry, suggesting that publication bias was acceptable and was unlikely to drive conclusions (Fig 6).

## Discussion

Totally laparoscopic gastrectomy is increasingly used since its debut in 1992 [26]. Several meta-analyses hinted totally laparoscopic distal gastrectomy (TLDG), pooling miscellaneous intracorporeal anastomosis including DA, BII and R-Y, has equivalent feasibility and safety as LADG [27–29]. More importantly, TLDG is seemingly less invasive and more suitable for the obese than LADG. Potential heterogeneity arises from different intracorporeal anastomosis in these studies and obscures the veritable efficacy of each method. To obtain a more reliable comparison on the feasible and safety of DA, we conducted this systematic review and meta-analysis of published articles that directly compared the DA with LADG BI.

There is no consensus on the indications of DA yet. The decision of DA usually resides with surgeons’ or patients’ preferences [13,20,23]. DA was mostly applied in early gastric cancer (EGC) located in the low third stomach, which combined indications of LG and conventional BI anastomosis [23,25]. Indications were also expanded to locally advanced gastric cancers (AGC) along with the accumulating surgeons’ experiences [20,21,24]. Remarkably, radical treatment of AGC usually requires longer surgical margin than EGC, which increases the anastomosis tension and results in related complications. Lin et al. reported patients with AGC underwent DA had a higher risk of anastomotic leakage [24]. Identifying the lesion precisely during the operation and achievement of enough surgical margin is crucial in deciding the methods of anastomosis. Distinguishing from LADG BI, DA is deficient with tactility in detecting the lesion of EGC merely by the laparoscopic grasper. Preoperative or intraoperative endoscopy is quite practical to mark the lesion as recommended by quite a few of surgeons [19,20].

Prolonged operation time is a common concern of laparoscopic surgery. Our meta-analysis revealed DA wasn’t inferior to LADG BI in this outcome. By using the liner stapler, DA has much saved the anastomosis time as well as the total operation time. In experienced hands, the operation time of LG with DA can be shorter than open gastrectomy and laparoscopy-assisted gastrectomy [13,20,23,24,30]. But this doesn’t mean the DA is easy. Conversely, coordination between the surgeon and assistants is especially technically demanding. During joining the posterior walls of duodenum and stomach together, appropriate traction by the assistant is of great importance. Given the majority of studies included in our meta-analysis were conducted by surgeons well-trained in LG, or even in DA, outcomes may be discrepant in the non-specially trained surgeons. Kanaya et al. and Jeong et al. revealed the learning curve of DA was quite steep for laparoscopic surgeons [23,30]. After around 10~15 cases, time of completing DA would reach a plateau and maintain in 10~25 min.

Comparing with LADG BI, the potential benefits of DA are better cosmesis, less blood loss and less postoperative pain. Usually, the surgical incision in LADG BI is longer than DA. By hiding the largest surgical incision around the umbilicus, patients undergo DA appears to be scarless. Avoiding the mini-laparotomy for gastrointestinal anastomosis, DA is totally intracorporeal and more tense-free, which reduces the risk of injury around the anastomosis. These characteristics of DA contribute to less intraoperative blood loss, less postoperative pain and more comfortable experience presenting as lower pain score and less administration of analgesics. In the studies by Kim et al. and Wang et al., postoperative pain score was significantly lower in DA than in the LADG BI[20,21]. Kinoshita et al. reported the acute inflammatory response following operation was milder in DA with a placid elevation of C-reactive protein [19]. In the present study, data from pooled studies were insufficient to evaluate the postoperative pain and acute inflammatory response. The evaluation of these parameters after DA will be sufficiently assessed by prospective RCTs only.

Acceptable postoperative complication of DA was observed in the present study, which was equivalent to LADG BI and even lower than open distal gastrectomy as historical reports [31,32]. Anastomosis-related complication such as leakage and stricture can be disastrous. There was a higher leakage rate in DA (DA *vs*. LADG BI, 10/755 vs.5/984), but it did not achieve a significant statistical difference. Kim et al. proposed mobilization of the duodenum until the gastroduodenal artery exposure to make sufficient duodenal stump, which eased the tension of anastomosis and might reduce the risk of anastomotic leakage [20]. DA contained several cutting edges, which may cause poor blood supply, yield more weak points around the anastomosis and result in leakage. Huang et al. modified the DA by resection the intersection of the duodenal cutting edge and the common closed edge at the same time to lessen the anastomotic weak point [33,34]. In contrast to leakage, DA group has less anastomotic stricture (2/755 vs.6/984). In LADG BI the size of anastomosis ring depends on the diameter of the circular stapler, while a large anastomosis ring can be easily achieved with the 45-mm linear stapler in DA. However, we should also admit the fact that the sample size was still too small to reveal the real benefits and drawbacks of DA because of the low risk of anastomotic leakage and stricture as reported.

Milder surgical trauma and comparable postoperative morbidity brought substantial clinical superiority. Patients in the DA group were able to resume oral intake earlier and have a shorter length of hospitalization, which could counteract the higher expense of DA technique itself. Enhanced recovery program was also applied in some included studies, which emphasized earlier oral intake and might contribute to these advantages of DA in some degrees. Apart from this, LADG BI had more severe complications demanded reoperation or other interventions, which postponed the discharge.

An unexpected result of our study was that the DA retrieved more lymph nodes than LADG BI. It didn’t mean the DA was superior to LADG BI in lymphadenectomy because this procedure is supposed to be identical for both approaches. We deemed surgeons’ experience played an important role as the majority of surgeons converted to DA after they had matured LADG BI [13,19]. Both DA and LADG BI achieved proximal resection margins more than 3cm that was believed to improve oncological outcomes[35]. Pooled analysis also demonstrated these two approaches achieved similar proximal surgical margins and overall survival rate.

Large anastomotic ring and straightforward passageway from the esophagus to the duodenum allow fast gastric emptying and permit more food intakes. More food intake and better nutritional status was observed in DA group during long-term follow-up [22]. Clinically significant postgastrectomy symptoms will deteriorate postoperative QoL. Without the barrier of the pylorus, large anastomosis ring of DA has the concern of reflux gastritis, which makes patients uncomfortable and increases in risk of gastric remnant cancer [36–38]. Against with this hypothesis, several studies reported the rate of reflux gastritis after DA was acceptable and comparable with conventional LADG BI and R-Y. Both Kanaya et al. and Lee et al. reported DA had a prevalence of bile reflux but was not a clinical problem [22,30]. Other symptoms are also concerned. Twisting duodenum after DA appears to cause gastric stasis and gastroesophageal reflux. On the other hand, twisting duodenum happened to ward off dumping syndrome in some degree. According to the follow-up result, DA had a similar risk of these symptoms as LADG BI as showed in Table 4.

This meta-analysis had several limitations which should be taken into consideration in interpreting the conclusions of this study. First, this meta-analysis pooled eight retrospective studies which may bias the interpretation of their results. Though well-designed randomized clinical trials are suitable for meta-analysis, RCTs on this issue are rarely conducted because of ethical concern or practical difficulty. Nevertheless, all eight pooled studies were of high quality according to the NOS. Abraham et al. have found that meta-analysis of the well-designed non-randomized clinical trial of surgical procedures was probably as accurate as that of RCTs [39]. Second, case volumes of the included studies varied greatly, which may lead to heterogeneity among studies. In such a case, comparisons of surgical results would be influenced by surgeons’ experience. Third, DA and LADG BI were performed in different periods, from 2004–2014. Due to the development of laparoscopic instruments, perioperative management protocol and surgeons’ surgical techniques, the clinical outcomes varied and may result in biases. Fourth, long-term functional outcomes in terms of nutritional status and postgastrectomy symptoms weren’t directly compared in the present meta-analysis because four pooled studies report using inconsistent assessment scales with different follow-ups. Furthermore, our study was based on studies conducted in East Asia which should extrapolate these data to the Western population prudently, in where the patient population and disease biology may differ [40,41].

## Conclusion

Our study suggests that the DA is a safe and feasible procedure as compared with LADG BI. DA broadens the options of gastrointestinal reconstruction following laparoscopic distal gastrectomy and might bring more minimally invasive benefits and better postoperative nutritional status. However, well designed large-scaled studies which balance the baseline of each arm are needed for further confirming the real benefits of DA.

## Supporting Information

S1 Text PRISMA Checklist. (DOC)

S2 Text Search Strategy. (DOC)
