# Supplementary material for: Comparison of Delta-Shape Anastomosis and Extracorporeal Billroth I Anastomosis after Laparoscopic Distal Gastrectomy for Gastric Cancer: A Systematic Review with Meta-Analysis of Short-Term Outcomes
Source: PLoS One. 2016 Sep 15;11(9):e0162720. doi: 10.1371/journal.pone.0162720 (PMC5025198; doi:10.1371/journal.pone.0162720)
Supplement: S2 Text — (DOC) [file pone.0162720.s002.doc]

The search strategy for the PubMed was：(((((((gastric neoplasms) OR gastric adenocarcinoma) OR gastric cancer)) AND ((minimally invasive gastrectomy) OR laparoscopic gastrectomy)) AND ((delta-shaped anastomosis) OR intracorporeal Billroth I anastomosis)) AND ( ( Chinese[lang] OR English[lang] ) )).

Similar search strategies were conducted in other databases.
